# Association between microbiome and the development of adverse posttraumatic neuropsychiatric sequelae after traumatic stress exposure

**DOI:** 10.1038/s41398-023-02643-8

**Published:** 2023-11-18

**Authors:** Abigail L. Zeamer, Marie-Claire Salive, Xinming An, Francesca L. Beaudoin, Stacey L. House, Jennifer S. Stevens, Donglin Zeng, Thomas C. Neylan, Gari D. Clifford, Sarah D. Linnstaedt, Scott L. Rauch, Alan B. Storrow, Christopher Lewandowski, Paul I. Musey, Phyllis L. Hendry, Sophia Sheikh, Christopher W. Jones, Brittany E. Punches, Robert A. Swor, Lauren A. Hudak, Jose L. Pascual, Mark J. Seamon, Erica Harris, Claire Pearson, David A. Peak, Roland C. Merchant, Robert M. Domeier, Niels K. Rathlev, Brian J. O’Neil, Paulina Sergot, Leon D. Sanchez, Steven E. Bruce, Ronald C. Kessler, Karestan C. Koenen, Samuel A. McLean, Vanni Bucci, John P. Haran

**Affiliations:** 1 Department of Microbiology and Physiologic Systems, University of Massachusetts Chan Medical School, Worcester, MA USA; 2 Department of Emergency Medicine, University of Massachusetts Chan Medical School, Worcester, MA USA; 3 Institute for Trauma Recovery, University of North Carolina at Chapel Hill, Chapel Hill, NC USA; 4 Department of Epidemiology, Brown University, Providence, RI USA; 5 Department of Emergency Medicine, Brown University, Providence, RI USA; 6 Department of Emergency Medicine, Washington University School of Medicine, St. Louis, MO USA; 7 Department of Psychiatry and Behavioral Sciences, Emory University School of Medicine, Atlanta, GA USA; 8 Department of Biostatistics, Gillings School of Global Public Health, University of North Carolina, Chapel Hill, NC USA; 9 Departments of Psychiatry and Neurology, University of California San Francisco, San Francisco, CA USA; 10 Department of Biomedical Informatics, Emory University School of Medicine, Atlanta, GA USA; 11 Department of Biomedical Engineering, Georgia Institute of Technology and Emory University, Atlanta, GA USA; 12 The Many Brains Project, Belmont, MA USA; 13 Department of Psychiatry, Harvard Medical School, Boston, MA USA; 14 Institute for Technology in Psychiatry, McLean Hospital, Belmont, MA USA; 15 Department of Psychiatry, McLean Hospital, Belmont, MA USA; 16 Department of Emergency Medicine, Vanderbilt University Medical Center, Nashville, TN USA; 17 Department of Emergency Medicine, Henry Ford Health System, Detroit, MI USA; 18 Department of Emergency Medicine, Indiana University School of Medicine, Indianapolis, IN USA; 19 Department of Emergency Medicine, University of Florida College of Medicine-Jacksonville, Jacksonville, FL USA; 20 Department of Emergency Medicine, Cooper Medical School of Rowan University, Camden, NJ USA; 21 Department of Emergency Medicine, Ohio State University College of Medicine, Columbus, OH USA; 22 Ohio State University College of Nursing, Columbus, OH USA; 23 Department of Emergency Medicine, Oakland University William Beaumont School of Medicine, Rochester, MI USA; 24 Department of Emergency Medicine, Emory University School of Medicine, Atlanta, GA USA; 25 Department of Surgery, University of Pennsylvania, Philadelphia, PA USA; 26 Perelman School of Medicine, University of Pennsylvania, Philadelphia, PA USA; 27 Department of Emergency Medicine, Einstein Medical Center, Philadelphia, PA USA; 28 Department of Emergency Medicine, Wayne State University, Ascension St. John Hospital, Detroit, MI USA; 29 Department of Emergency Medicine, Massachusetts General Hospital, Boston, MA USA; 30 Department of Emergency Medicine, Brigham and Women’s Hospital, Boston, MA USA; 31 Department of Emergency Medicine, Trinity Health-Ann Arbor, Ypsilanti, MI USA; 32 Department of Emergency Medicine, University of Massachusetts Medical School-Baystate, Springfield, MA USA; 33 Department of Emergency Medicine, Wayne State University, Detroit Receiving Hospital, Detroit, MI USA; 34 Department of Emergency Medicine, McGovern Medical School at UTHealth, Houston, TX USA; 35 Department of Emergency Medicine, Harvard Medical School, Boston, MA USA; 36 Department of Psychological Sciences, University of Missouri - St. Louis, St. Louis, MO USA; 37 Department of Health Care Policy, Harvard Medical School, Boston, MA USA; 38 Department of Epidemiology, Harvard University, Boston, MA USA; 39 Department of Emergency Medicine, University of North Carolina at Chapel Hill, Chapel Hill, NC USA; 40 Program in Microbiome Dynamics, University of Massachusetts Chan Medical School, Worcester, MA USA

**Keywords:** Human behaviour, Depression

## Abstract

Patients exposed to trauma often experience high rates of adverse post-traumatic neuropsychiatric sequelae (APNS). The biological mechanisms promoting APNS are currently unknown, but the microbiota-gut-brain axis offers an avenue to understanding mechanisms as well as possibilities for intervention. Microbiome composition after trauma exposure has been poorly examined regarding neuropsychiatric outcomes. We aimed to determine whether the gut microbiomes of trauma-exposed emergency department patients who develop APNS have dysfunctional gut microbiome profiles and discover potential associated mechanisms. We performed metagenomic analysis on stool samples (n = 51) from a subset of adults enrolled in the Advancing Understanding of RecOvery afteR traumA (AURORA) study. Two-, eight- and twelve-week post-trauma outcomes for post-traumatic stress disorder (PTSD) (PTSD checklist for DSM-5), normalized depression scores (PROMIS Depression Short Form 8b) and somatic symptom counts were collected. Generalized linear models were created for each outcome using microbial abundances and relevant demographics. Mixed-effect random forest machine learning models were used to identify associations between APNS outcomes and microbial features and encoded metabolic pathways from stool metagenomics. Microbial species, including *Flavonifractor plautii*, *Ruminococcus gnavus* and, *Bifidobacterium* species, which are prevalent commensal gut microbes, were found to be important in predicting worse APNS outcomes from microbial abundance data. Notably, through APNS outcome modeling using microbial metabolic pathways, worse APNS outcomes were highly predicted by decreased L-arginine related pathway genes and increased citrulline and ornithine pathways. Common commensal microbial species are enriched in individuals who develop APNS. More notably, we identified a biological mechanism through which the gut microbiome reduces global arginine bioavailability, a metabolic change that has also been demonstrated in the plasma of patients with PTSD.

## Introduction

Adverse posttraumatic neuropsychiatric sequelae (APNS) such as posttraumatic stress disorder (PTSD), depression, and somatic symptoms are common after traumatic stress exposure [1–6]. Contemporary limitations in understanding the pathogenesis of APNS are a barrier to developing effective primary and secondary preventive interventions [1, 7]. Novel multidisciplinary approaches applying methodologic advances from other areas of neuroscience to the early post-trauma period may advance understanding of APNS pathogenesis.

One such area is the study of the microbiome-gut-brain axis. The microbiome-gut-brain axis has been demonstrated to have an important influence on brain function in neuropsychiatric disorders [8–10], and has been hypothesized to play an important role in trauma-related neuropsychiatric disorders [11]. Gut microbes affect central nervous system function through the production of metabolites and neurochemicals [10]. For example, short-chain fatty acids such as butyrate support gut homeostasis by maintaining gut barrier integrity and inducing anti-inflammatory factors which cross the blood-brain barrier [12, 13]. In contrast, *Enterobacteriaceae* expansion can augment neuroinflammatory processes through the lipopolysaccharide (LPS) endotoxin-mediated activation of the Toll-like receptor 4 (TLR4) pathway, leading to the production of pro-inflammatory cytokines [14].

While specific mechanisms responsible for it remain incompletely understood, dysregulated immunity and elevated inflammation are risk factors for PTSD [15]. Given the above known influence of the gut-brain axis on neuroinflammation, these data support the hypothesis that variations in microbiome characteristics could influence APNS pathogenesis. This hypothesis is also supported by several small studies which identified broad phylum-level differences in microbiome characteristics among individuals with PTSD (*n* = 18) as compared to those without PTSD (*n* = 12) [16], and in veterans which found associations between lower diversity and a higher abundance of opportunistic microbes with PTSD symptoms and impaired cognition [17]. This hypothesis is also supported by animal studies in which trauma was found to cause alterations in the microbiome and lead to increased local and systemic inflammation [8].

In this nested study, we explored the association between the gut microbiome and APNS development in a subsample of individuals recruited from emergency departments (ED) in the immediate aftermath of trauma as part of the AURORA study (*n* = 74) and who also provided stool samples [1]. We employed shotgun metagenomic sequencing of stool samples from patients evaluated and discharged from the ED and machine learning (ML) predictive analytics to test the hypothesis that variability in microbiome species and pro- and anti-inflammatory metabolic pathways is associated with APNS outcomes.

## Patients and Methods

### Study Population and Sample Collection

The AURORA study began in September 2017; we pre-registered a nested microbiome pilot study with the AURORA consortium and enrolled participants from November 2020 until February 2021 (Supplemental table 1) [18]. AURORA participants who completed their first outpatient remote assessment and had not received antibiotics within the previous 6 months were approached for enrollment into this nested study. Participants also could not have had any other influences that could cause major microbiome perturbations (e.g., infections). Of the 2,097 AURORA participants subjects approached, 106 provided consent to participate, and we received stool samples from *n* = 74 of these individuals (69.8%). Stool samples were self-collected at home using OMNIgene•GUT collection kits (DNAgenotek, catalog no. OMR-200). All stool samples were self-collected at least five days after the initial ED presentation (Supplemental figure 1). Upon receipt, samples were stored at -80 °C until DNA extraction and sequencing were performed. This study was approved by the University of North Carolina’s institutional review board (IRB protocol #17-0703) and by the UMass Chan Medical School’s institutional review board.

### Outcome Measurements

After receiving written informed consent from eligible patients, study coordinators from each participating ED performed data collection for ED-based assessments, including baseline questionnaires. Follow-up evaluations were internet-based at the two-, eight-, and twelve-week intervals. We assessed posttraumatic stress symptoms using the Post Traumatic Stress Disorder (PTSD) checklist for DSM-5 (PCL-5) [19–22]. Per this instrument’s guidelines, a PTSD diagnosis can be made by summing the self-reported responses to the PCL-5. We used total sum of 31 for a positive diagnosis of PTSD [23]. A normalized t-score for depression severity and the diagnosis was assessed using the PROMIS Depression Short Form 8b [24]. A t-score of 60 is one standard deviation lower than average and was used to designate a depression diagnosis. The Rivermead Post-concussive questionnaire (RPQ) was used to obtain a count of somatic symptoms. The twelve post-traumatic somatic symptoms included in the RPQ are headache, dizziness, nausea, noise sensitivity, fatigue, insomnia, poor concentration, taking longer to think, blurred vision, light sensitivity, double vision, and restlessness [25]. For each symptom, a yes/no variable was created. A positive response to a symptom would be counted as YES = 1 that it does exist, while a NO = 0 indicates that the symptom does not exist. The yes/no responses were summed to provide a simple count of somatic symptoms.

### Sample Processing and DNA Sequencing

Prior to extraction, stool samples were heat inactivated at 65 °C-70 °C for 1 hour and stored at -80 °C. Approximately 250 mg of resulting sample was extracted using the QIAGEN DNeasy PowerSoil Pro Kits (QIAGEN, catalog no. 47016). Sequencing libraries were prepped using the Nextera XT DNA library prep kit and sequenced on a NextSeq 500 sequencer with 2×150 bp paired-end reads. Of the 74 stool samples received, 69 (93.2%) were successfully sequenced. Samples with a percentage of reads identified greater than 0.5%, and complete metadata were used for analysis (*n* = 51).

Shotgun metagenomic reads were trimmed and filtered for host contamination using the KneadData pipeline (https://github.com/biobakery/kneaddata). The resulting metagenomic data was profiled for microbial abundance and metabolic pathways using the MetaPhlAn3 [26] and HUMAnN3 databases and tools [27]. The resulting relative microbial species abundance and metabolic pathway data was used for downstream analysis in R.

### Microbiome Analysis and Statistics

#### Power Analysis

We performed a power analysis to estimate the study size according to a one sample Spearman correlation. Spearman correlation is a non-parametric test and is thus suitable for microbiome data. With a sample size of 51 a correlation of 0.5 (a medium effect size), the power was found to be 0.97. This analysis was performed in R using the pwr package (https://github.com/heliosdrm/pwr).

#### Linear mixed model-based analysis to determine the relative contribution of the microbiome

To determine how much of the variability in post-trauma APNS outcomes is explained by the gut microbiome, we first constructed linear mixed models (LMM) using the lme4 R package [28] for each of the three APNS outcomes of interest (PTSD, Depression Scores and Somatic Symptoms) as a function of relevant clinical covariates (sex, age, body mass index, race/ethnicity) [29], and also of the arcsine square root-transformed abundance [30] of each microbial species independently. Features identified as significant (FDR corrected p-value <= 0.05) were combined and used to fit a global LMM. The contribution of each feature to the regression line fit by the model was determined by running analysis of variance (ANOVA) and examining the total sum of squares for the fixed effects. The resulting sum of squares ratio for each feature was graphed using WebR (https://github.com/cardiomoon/webr).

#### Mixed-effect random forest analysis of microbiome permutated importance to outcomes at twelve weeks post-trauma

Microbiome data is non-linear and not normally distributed [31, 32]. Additionally, such data are characterized by many predictors, which, if combined in the same model (without prefiltering), can prohibit a traditional regression model to converge [33]. We have previously demonstrated that tree-based machine learning (ML) approaches such as random forest, which are non-parametric and perform intrinsic feature selection, are very powerful in finding a signal from static and time-resolved microbiome data [34–37]. To fully utilize the longitudinal clinical data collected by the AURORA (parent) study for this nested study [1], we assumed, barring perturbation, the gut microbiome is stable over time. Several high-resolution gut microbiome temporal studies have demonstrated that the microbiome is rather stable and displays only small random fluctuations [38–41]. The extent to which the microbiome is stable in individuals that have experienced trauma remains to be established. We built a mixed-effect random forest (MERF) regression model to predict either PTSD raw score, Depression t-score, or somatic symptoms count at twelve weeks post-trauma. We use either microbial abundance or metabolic pathway abundance and relevant clinical covariates (e.g., BMI, age, sex, and race) as variables in this modeling. The first step of our pipeline split our data into a training and test set. To predict twelve-week post-trauma outcomes, two-week and eight-week post-trauma outcomes were used to train the MERF model. The unseen twelve-week data was used for testing the model. For each APNS outcome, the pipeline was run ten times with ten different random seeds, and model performance, statistics, and outcomes were calculated for each seed. Model performance was evaluated by Root Mean Square Error (RMSE) and correlation of true versus predicted values, which illustrates the model’s accuracy and fit for predicting outcome measurements at twelve weeks post-trauma (Supplemental figure 2). Permuted variable importance was calculated and used to evaluate models. Plots summarizing results were generated in R using the ggplot2.

## Results

### Characteristics of Study Subjects

Among 2,097 individuals who were approached in the ED regarding this nested study, 106/2,097 (5.1%) agreed to participate, and 74/106 (69.8%) provided a stool sample. Of the stool samples received, 51(68.9%) passed all quality and metadata checks (see methods) and were profiled for microbial abundance and metabolic pathways. From these 51 participants (mean age 52 years; 26 (51%) female) stool samples were received a median of 45 days (range 5 to 182 days) (Supplemental figure 1) after ED visit for trauma evaluation that conferred entry to the AURORA (parent) study (Table 1). The most common types of trauma were motor vehicle collision (55%, *n* = 28), fall from height (*n* = 9, 18%), and other accidental or targeted/involuntary events (9.8%, *n* = 5). Stool samples originated primarily from White (61%, *n* = 31) and Black (25%, *n* = 13) participants. Of the 31 white participants, 29 (94%) were non-Hispanic White. Microbiome diversity measures show that individuals do not stratify based on alpha (Simpson and Shannon) or beta diversity.

**Table 1:** Patient Characteristics by APNS outcome category.

		Depression			PTSD		
Patient Characteristics	Overall, N = 51^a^	Depression-, N = 35^a^	Depression + , N = 16^a^	q-value (adjusted p-value^b^)^c^	PTSD-, N = 31^a^	PTSD + , N = 20^a^	q-value (adjusted p-value^b^)^c^
**Demographics**							
Age in years (at enrollment)	52 (36, 60)	52 (36, 59)	52 (37, 60)	>0.9	52 (36, 58)	50 (34, 61)	>0.9
Sex (Female)	26 (51%)	19 (54%)	7 (44%)	>0.9	19 (61%)	7 (35%)	0.7
BMI	29 (24, 33)	27 (24, 30)	33 (29, 40)	0.11	27 (23, 30)	31 (29, 36)	0.083
White	31 (61%)	21 (60%)	10 (62%)	>0.9	18 (58%)	13 (65%)	>0.9
Black	13 (25%)	11 (31%)	2 (12%)	>0.9	9 (29%)	4 (20%)	>0.9
Asian	1 (2.0%)	1 (2.9%)	0 (0%)	>0.9	1 (3.2%)	0 (0%)	>0.9
other	6 (12%)	2 (5.7%)	4 (25%)	0.8	3 (9.7%)	3 (15%)	>0.9
Hispanic	7 (14%)	4 (11%)	3 (19%)	>0.9	4 (13%)	3 (15%)	>0.9
**U.S. Geographic Region**				>0.9			>0.9
Midwest Region	10 (20%)	5 (14%)	5 (31%)		5 (16%)	5 (25%)	
Northeast Region	38 (75%)	27 (77%)	11 (69%)		24 (77%)	14 (70%)	
Southern Region	3 (5.9%)	3 (8.6%)	0 (0%)		2 (6.5%)	1 (5.0%)	
**Trauma Event Type (Broad)**				>0.9			>0.9
Animal-related	1 (2.0%)	1 (2.9%)	0 (0%)		1 (3.2%)	0 (0%)	
Fall < 10 feet or from unknown height	9 (18%)	7 (20%)	2 (12%)		6 (19%)	3 (15%)	
Fall >= 10 feet	4 (7.8%)	2 (5.7%)	2 (12%)		2 (6.5%)	2 (10%)	
Incident causing traumatic stress exposure to many people	1 (2.0%)	1 (2.9%)	0 (0%)		1 (3.2%)	0 (0%)	
Motor Vehicle Collision	28 (55%)	19 (54%)	9 (56%)		17 (55%)	11 (55%)	
Non-motorized Collision	3 (5.9%)	2 (5.7%)	1 (6.2%)		2 (6.5%)	1 (5.0%)	
other	5 (9.8%)	3 (8.6%)	2 (12%)		2 (6.5%)	3 (15%)	
Self-Reported Perceived Chance of Dying	6.0 (1.0, 8.5)	6.0 (1.0, 8.5)	5.5 (1.8, 8.2)	>0.9	6.0 (0.5, 8.5)	5.5 (1.8, 8.5)	>0.9

^a^n (%); Median (IQR)

^b^Pearson’s Chi-squared test; Fisher’s exact test; Wilcoxon rank sum test

^c^Bonferroni correction for multiple testing

### Gut Microbiome Features associated with APNS

**Fig. 1:**
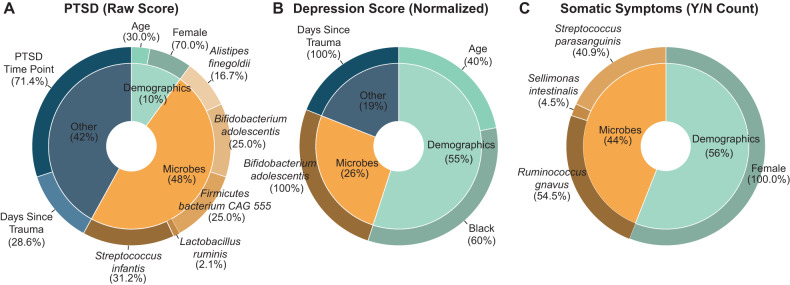
Contribution of microbiome features significantly associated with neuropsychiatric outcomes. After metagenomic sequencing, microbial species abundances were combined with demographics and select clinical variables (other) in linear mixed effect models for the APNS outcomes at two-, eight-, and twelve weeks post trauma (**A**) PTSD raw score as determined by the DSM-5 PTSD checklist, (**B**) depression score as determined by the PROMIS Depression Short Form 8b and (**C**) somatic symptoms count (Yes/No) based on the Rivermead Post-Concussive Questionnaire. Individual microbial species, demographics, and clinical variables found as significant are displayed in the outer wheels. PTSD time point reflects the time at which the modeled score was taken (either two weeks, eight weeks, or twelve weeks). The length of time between trauma exposure and stool sample collection was included as days since trauma in all models.

Linear mixed effect models and ANOVA adjusting for sociodemographic characteristics were used to assess for associations between microbiome characteristics and APNS symptom severity. Gut microbiome characteristics accounted for 48%, 26%, and 44% of the variation in PTSD, depressive, and somatic symptoms after trauma, respectively (Fig. 1). The abundance of *Firmicutes bacterium CAG:555, Bifidobacterium adolescentis*, and the pro-inflammatory *Streptococcus infantis* were associated with PTSD symptom severity. The abundance of *B. adolescentis* was associated with depressive symptom severity (Fig. 1B). *Ruminococcus gnavus* and *Streptococcus parasanguinus* were associated with somatic symptom severity (Fig. 1C).

### Post-trauma neuropsychiatric outcomes are predicted by microbial abundance

While the above LMM approach is useful to assess total variance accounted for by microbiome characteristics and is common in the field, it has several limitations, including the inability to evaluate microbial abundances in the context of the entire microbiome and the limited ability to account for inter-individual microbiome differences [33]. Therefore, we used a complementary machine learning approach to train and test models examining associations between gut microbiome characteristics and posttraumatic outcomes. We have previously used this approach and have determined its optimality for inferring biologically relevant host-microbe interactions from cross-sectional and longitudinal microbiome data (see methods sections for further justification) [34–37, 42–44].

**Fig. 2:**
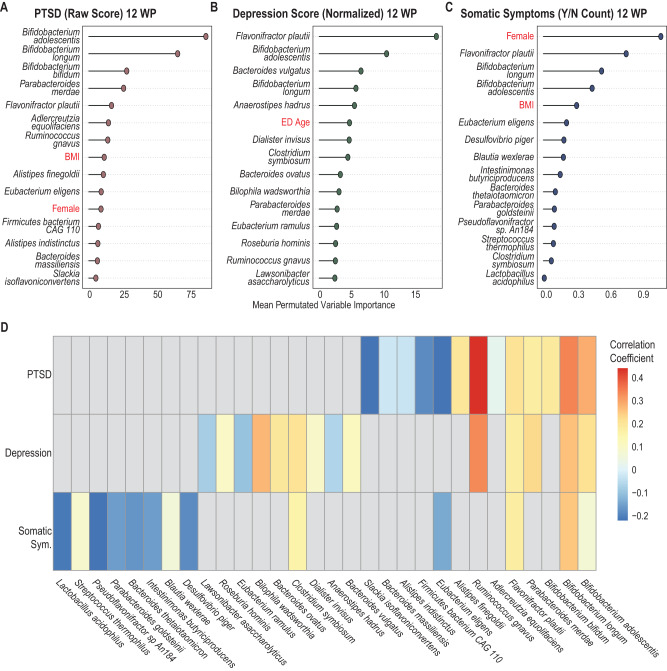
Mixed-Effect Random Forest (MERF) Regression Models Using Microbial Abundance and Clinical Covariates to Predict Neuropsychiatric Outcomes. MERF models combining microbial abundance data with clinical and demographic (highlighted in red) features demonstrate the importance of microbial features in predicting outcomes. Permutated importance analysis of model outcomes shows the top 15 features contributing to predictions of (**A**) PTSD raw score, (**B**) depression normalized score, and (**C**) somatic symptoms count (yes/no) are mostly microbial species. **D** Heat map showing correlation coefficients of the top 30 contributing species show common species that contribute to all three outcomes (increased abundance) and species which associate with individual outcomes (mostly decreased abundance).

A Mixed-Effect Random Forest (MERF) regression model was used to identify microbiome features associated with PTSD, depression, and somatic symptom burden at twelve weeks post-trauma (Supplemental figure 2). Our findings using this approach confirmed and expanded on findings from the linear mixed-effect models. Bacterial species identified in linear mixed effects modeling, specific species of the *Alistipes, Bifidobacterium*, and *Ruminococcus* genera were also found as important predictors by the MERF model (Fig. 2). The models also identified more species as contributors to each outcome, with many similar species in the top 15 predictors identified by the three models. *B. adolescentis*, *B. longum*, and *Flavonifractor plautii* were among the top five predictors for all three APNS outcomes (Fig. 2). Across our models, both *Bifidobacterium* species and *F. plautii* showed similar trends of increased abundance being informative of higher scores (Supplemental figure 3). *Ruminococcus gnavus* was found to be among the top 15 predictors only for PTSD (ranked 7^th^) and depression (ranked 14^th^), with increases in abundance correlating with disease outcomes (Fig. 2, Table 2). Decreases in certain species were noted to cluster by individual outcomes for the most part (Fig. 2D).

**Table 2:** Spearman’s Correlation of Important Microbes with APNS Outcomes.

Predictors	PTSD	Depression	Somatic Symptoms	Notes
*Bifidobacterium adolescentis*	0.286	0.216	0.0704	• Produces GABA in the gut [69] • Induction of Th17 cells in mice [70] • Decreased in Crohn’s Disease [71] • Lower abundance in children with autism spectrum disorder [72]
*Bifidobacterium longum*	0.34	0.259	0.251	• GABA production in vivo [73] • Anxiolytic effect through the vagus nerve in mice [57] • Lower abundance in children with autism spectrum disorder [72] • Strain specific induction of cytokines in peripheral blood mononuclear cells [74]
*Bifidobacterium bifidum*	0.185	*NA*	*NA*	• Th17 inducing profile [75] • Lower abundance in children with autism spectrum disorder [72]
*Parabacteroides merdae*	0.176	0.226	*NA*	• Indole negative [76] • Rarely associated with infections[77] • Branched-chain amino acid catabolism by *P. merdae* reduces atherosclerotic lesions [78]
*Flavonifractor plautii*	0.207	0.177	0.173	• In mice, suppresses Th2 immune response [79] • [80] • Genera found to be enriched in those with active major depressive disorder (MDD) [63]
*Adlercreutzia equolifaciens*	0.0195	*NA*	*NA*	• Strain specific equol production [81]
*Ruminococcus gnavus*	0.441	0.335	*NA*	• Increased in Crohn’s Disease [71] • Depleted genera level in active MDD [63]
*Alistipes finegoldii*	0.198	*NA*	*NA*	• Can hydrolyze tryptophan to indole; Succinic acid producer in vitro with minor acetic and propionic acid; bile-resistant, esculin-negative; catalase-negative; nitrogen-reductase negative [82, 83] • Enriched in colorectal cancer [84] • Main cellular fatty acid 13-metyltetradecanoic acid (iso-C15:0) • Genera Increased abundance in active MDD patients compared to healthy controls [63]
*Eubacterium eligens*	−0.216	*NA*	-0.14	• Decreased in COVID-19 health care workers with higher stress scores [49] • Anti-inflammatory related [85]
*Firmicutes bacterium CAG 110*	−0.182	*NA*	*NA*	• Identified in livestock [86]
*Alistipes indistinctus*	−0.0339	*NA*	*NA*	• Unable to hydrolyze tryptophan to indole; susceptible to bile; catalase-positive; urease and nitrogen reductase-negative; Succinic and acetic acid producer in vitro [83] • Main cellular fatty acid 13-metyltetradecanoic acid (iso-C15:0) • Genera Increased abundance in active MDD patients compared to healthy controls [63]
*Bacteroides massiliensis*	−0.0271	*NA*	*NA*	• *Phocaeicola massiliensis* • Genera increased in health controls vs. active MDD patients [63]
*Slackia isoflavoniconvertens*	−0.221	NA	*NA*	• Capable of equol production [87]
*Desulfovibrio piger*	*NA*	*NA*	−0.183	• Sulfur-reducing bacteria associated with inflammatory bowel disease [88]
*Blautia wexlerae*	*NA*	*NA*	0.0584	• Inversely correlated with obesity and type 2 diabetes mellitus [89] • Decreased abundance in progressive multiple sclerosis [90]
*Intestinimonas butyriciproducens*	*NA*	*NA*	−0.155	• Butyrate production [91]
*Bacteroides thetaiotaomicron*	*NA*	*NA*	−0.172	• Genera increased in healthy controls vs. active MDD patients [63]
*Parabacteroides goldsteinii*	*NA*	*NA*	−0.155	• Anti-inflammatory and decreased in chronic inflammatory diseases including chronic obstructive pulmonary disease (COPD; negatively associates with severity) [92] and chronic kidney disease [93]
*Pseudoflavonifractor sp An184*	*NA*	*NA*	−0.215	• Positively associated with weight loss [94]
*Streptococcus thermophilus*	*NA*	*NA*	0.0866	
*Clostridium symbiosum*	*NA*	0.204	0.163	• Increased abundance in early colorectal cancer [95]
*Lactobacillus acidophilus*	*NA*	*NA*	−0.21	
*Bacteroides vulgatus*	*NA*	0.115	*NA*	• Genera increased in healthy controls vs. active MDD patients [63]
*Anaerostipes hadrus*	*NA*	−0.0635	*NA*	
*Dialister invisus*	*NA*	0.0954	*NA*	• [80] • Genera increased in healthy controls vs. active MDD patients [63]
*Bacteroides ovatus*	*NA*	0.194	*NA*	
*Bilophila wadsworthia*	*NA*	0.274	*NA*	
*Eubacterium ramulus*	*NA*	−0.096	*NA*	
*Roseburia hominis*	*NA*	0.102	*NA*	
*Lawsonibacter asaccharolyticus*	*NA*	−0.0814	*NA*	

### Metabolic Pathway Profiling Identifies Novel Gut-Brain-Axis Interface with APNS Outcomes

**Fig. 3:**
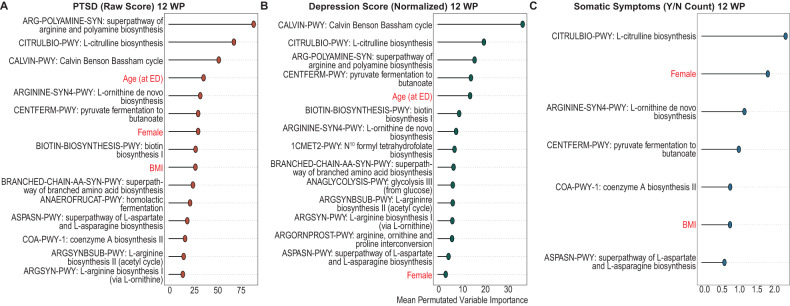
Mixed-Effect Random Forest (MERF) Regression Models Using Microbial Metabolic Pathways and Clinical Covariates to Predict Neuropsychiatric Outcomes. MERF models combining microbial metabolic pathway abundance data with clinical and demographic (highlighted in red) features demonstrate the importance of microbial pathways in predicting outcomes for (**A**) PTSD raw score, (**B**) depression normalized score, and (**C**) somatic symptoms count (yes/no). The top 15 features from analysis of permutated importance on model outcomes are displayed for PTSD and depression. Our model found only seven significant features for somatic symptoms.

**Fig. 4:**
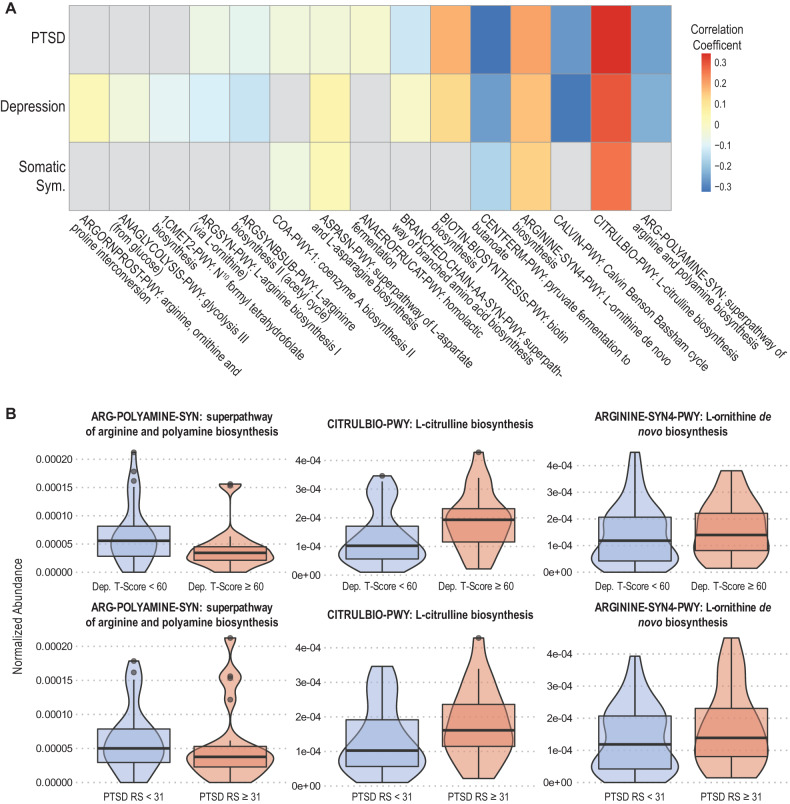
Important metabolic pathways for PTSD and depression involve arginine, citrulline and ornithine. Heat map (**A**) of correlation coefficients for the top 15 metabolic pathways contributing to PTSD, depression, and somatic symptoms from MERF analysis show significant contributions by amino acid and polyamine biosynthesis pathways. **B** Violin plots showing differences in contributions of arginine, citrulline, and ornithine biosynthesis pathways for depression (top graphs) and PTSD (bottom graphs). Blue violins indicate no diagnosis (PTSD raw score ≤ 31; Depression T-Score < 60 indicating none to mild depression) red indicates PTSD or depression diagnosis (PTSD RS > 31; Depression T-Score ≥ 60 indicating moderate to severe depression).

**Fig. 5:**
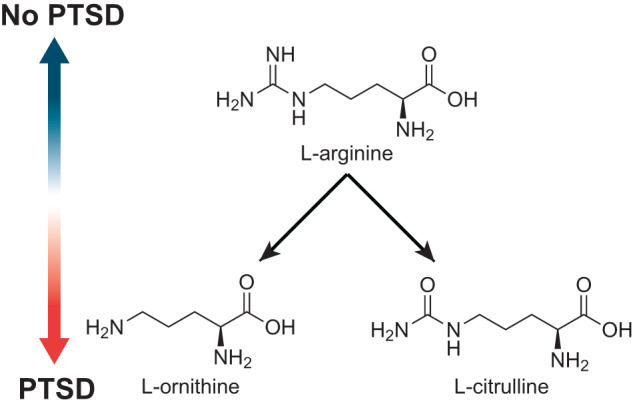
Arginine is converted into citrulline and ornithine commonly in the host and the microbiome. The ratio of arginine to citrulline and ornithine in PTSD patients has previously been found to negatively correlated with PTSD. We find that this negative correlation in the metabolic pathways encoded for in the microbiome of trauma survivors.

Species abundance, although informative of outcomes, does not directly provide context for the functional roles of associated microbes. By analyzing metabolic pathway abundances encoded in the metagenomic data, we can gain insight into the microbial products, metabolites, and functions that may associate with each outcome of interest, thus shedding light on possible mechanistic links. To this end, we employed the same MERF-based pipeline to examine associations between the abundance of microbially-encoded metabolic pathways and PTSD, depressive, and somatic symptom severity twelve weeks after trauma (Supplemental figure 4). The Calvin-Benson-Bassham cycle, a common CO_2_ fixation pathway in autotrophic bacteria, was identified as one of the top three predictors of PTSD and depressive symptom severity (i.e., cycle was reduced in samples with higher scores (Fig. 3A and B)). Amino acid biosynthesis pathways were a leading predictor for all three APNS outcomes, with the L-citrulline biosynthesis pathway identified as one of the top two predictors for all APNS outcomes. The super pathway of arginine and polyamine biosynthesis was identified as the top predictor for the PTSD model and as the third most important pathway for the depression model, correlating negatively with PTSD and depression scores (Fig. 4, Table 3). Furthermore, all three models identified pathways involving arginine, ornithine, and citrulline, three amino acids that are often interconverted (Fig. 5). De novo biosynthesis of L-ornithine, which can be interconverted from ornithine and urea to arginine and water, was identified by all models and appeared increased in patients with higher scores. Increased abundance of L-citrulline biosynthesis was also associated with higher scores of PTSD, depression, and somatic symptoms count (Fig. 4).

**Table 3:** Spearman’s Correlation of Important Metabolic Pathways with Outcomes.

Predictors	PTSD	Depression	Somatic Symptoms	Notes
ARG-POLYAMINE-SYN: superpathway of arginine and polyamine biosynthesis	−0.251	−0.229	*NA*	• Enriched patients with Crohn’s Disease (CD) in remission [96]
CITRULBIO-PWY: L-citrulline biosynthesis	0.346	0.298	0.261	• Enriched patients with CD in remission [96]
CALVIN-PWY: Calvin Benson Bassham cycle	−0.269	−0.315	*NA*	• Enriched patients with CD in remission [96]
ARGININE-SYN4-PWY: L-ornithine de novo biosynthesis	0.201	0.165	0.139	
CENTFERM-PWY: pyruvate fermentation to butanoate	−0.322	−0.26	-0.168	
BIOTIN-BIOSYNTHESIS-PWY: biotin biosynthesis I	0.181	0.127	*NA*	
BRANCHED-CHAIN AA-SYN-PWY: superpathway of branched amino acid biosynthesis	−0.127	−0.00818	*NA*	
ANAEROFRUCAT-PWY: homolactic fermentation	−0.0199	*NA*	*NA*	
ASPASN-PWY: superpathway of L-aspartate and L-asparagine biosynthesis	−0.0404	0.0571	0.037	
COA-PWY-1: coenzyme A biosynthesis II	−0.0454	*NA*	-0.053	
ARGSYNBSUB-PWY: L-arginine biosynthesis II (acetyl cycle)	−0.0768	−0.131	*NA*	
ARGSYN-PWY: L-arginine biosynthesis I (via L-ornithine)	−0.0579	−0.105	*NA*	
1CMET2-PWY: N^10^ formyl tetrahydrofolate biosynthesis	*NA*	−0.0885	*NA*	• Depleted in Ulcerative Colitis (UC) in remission [96]
ANAGLYCOLYSIS-PWY: glycolysis III (from glucose)	*NA*	−0.048	*NA*	
ARGORNPROST-PWY: arginine, ornithine and proline interconversion	*NA*	0.0264	*NA*

Given the strength of findings around pathways affecting global arginine levels, we next sought to identify microbes responsible for changes in these pathways. Focusing on ornithine, citrulline, and arginine, we examined the contribution of individual genera to each pathway (Tables 4, 5, 6) to PTSD and depression. Somatic symptoms were not further analyzed as the simple count nature of the variable was not appropriate for this analysis. We observed that in individuals with PTSD or depression, *Escherichia* had an increased contribution to all pathways involving arginine and ornithine biosynthesis. However, the contribution of *E. coli* to L-citrulline biosynthesis was decreased in those with PTSD and increased in those with depression. In patients with PTSD or depression, the *Ruminococcus* genus had a decreased contribution to L-arginine biosynthesis via both L-ornithine and the acetyl cycle. *Alistipes*, *Flavonifractor* and *Faecalibacterium* genera contributed more to the L-arginine biosynthesis via ornithine in individuals with diagnosed PTSD or depression.

**Table 4:** Microbial genus contribution to arginine-related metabolic pathways stratified by PTSD and depression diagnoses.

Pathway	Genus	PTSD Raw Score < 31	PTSD Raw Score ≥ 31	Depression t-score < 60	Depression t-score ≥ 60
ARG-POLYAMINE-SYN: Superpathway of arginine and polyamine biosynthesis	*Escherichia*	*53.64*	*69.95*	*42.86*	*100.00*
	*Klebsiella*	46.36	30.05	57.14	0.00
ARGSYNBSUB-PWY: L-arginine biosynthesis II (acetyl cycle)	*Akkermansia*	7.72	2.36	7.61	1.20
	*Anaerostipes*	2.49	0.67	2.29	0.68
	*Anaerotignum*	*0.36*	*1.14*	*0.48*	*1.03*
	*Bifidobacterium*	*1.07*	*1.47*	*1.01*	*1.72*
	*Bilophila*	*0.52*	*1.61*	*0.58*	*1.75*
	*Blautia*	4.12	4.00	4.46	3.11
	*Escherichia*	*0.97*	*3.81*	*1.04*	*4.37*
	*Faecalibacterium*	*33.79*	*41.04*	*32.37*	*46.62*
	*Flavonifractor*	*0.79*	*3.32*	*1.55*	*2.06*
	*Fusicatenibacter*	8.76	4.57	8.00	5.40
	*Gemmiger*	10.68	7.95	10.19	8.46
	*Klebsiella*	*0.96*	*1.74*	*1.73*	*0.00*
	*Lachnospiraceae unclassified*	*9.40*	*8.91*	*9.14*	*9.46*
	*Roseburia*	1.42	1.15	1.58	0.68
	*Ruminococcaceae unclassified*	2.43	1.16	2.55	0.50
	*Ruminococcus*	4.14	3.45	4.09	3.39
	*Tyzzerella*	*0.00*	*1.40*	*0.33*	*0.94*

**Table 5:** Microbial genus contribution to ornithine-related metabolic pathways stratified by PTSD and depression diagnoses.

Pathway	Genus	PTSD Raw Score < 31	PTSD Raw Score ≥ 31	Depression t-score < 60	Depression t-score ≥ 60
ARGININE-SYN4-PWY: L-ornithine de novo biosynthesis	*Bacteroides*	*38.78*	*29.26*	*37.40*	*28.63*
	*Catenibacterium*	*13.22*	*0.14*	*7.15*	*4.90*
	*Escherichia*	12.28	25.78	12.20	29.42
	*Klebsiella*	*5.21*	*4.50*	*8.41*	*0.00*
	*Parabacteroides*	28.82	35.58	33.49	31.06
	*Paraprevotella*	1.68	4.75	1.35	5.99
ARGSYN-PWY: L-arginine biosynthesis I (via L-ornithine)	*Alistipes*	3.21	16.69	3.92	18.18
	*Anaerostipes*	*2.60*	*0.38*	*2.40*	*0.31*
	*Bifidobacterium*	1.28	1.47	1.21	1.65
	*Blautia*	*3.95*	*3.29*	*4.29*	*2.46*
	*Escherichia*	1.36	3.40	1.45	3.65
	*Faecalibacterium*	38.95	39.19	37.33	42.50
	*Flavonifractor*	0.72	2.79	1.56	1.56
	*Fusicatenibacter*	*9.18*	*3.56*	*8.30*	*4.12*
	*Gemmiger*	*11.38*	*7.32*	*10.89*	*7.41*
	*Klebsiella*	0.55	0.77	0.96	0.00
	*Lachnospiraceae unclassified*	*9.42*	*7.48*	*9.13*	*7.64*
	*Roseburia*	*2.06*	*1.20*	*2.30*	*0.55*
	*Ruminococcaceae unclassified*	*2.32*	*1.02*	*2.49*	*0.41*
	*Ruminococcus*	*4.51*	*3.05*	*4.43*	*2.89*

**Table 6:** Microbial genus contribution to citrulline-related metabolic pathways stratified by PTSD and depression diagnoses.

Pathway	Species	PTSD Raw Score < 31	PTSD Raw Score ≥ 31	Depression t-score < 60	Depression t-score ≥ 60
CITRULBIO-PWY: L-citrulline biosynthesis	*Alistipes finegoldii*	61.07	70.93	54.58	76.92
	*Coprococcus catus*	2.81	0.00	1.92	0.00
	*Escherichia coli*	17.97	15.87	15.87	16.77
	*Flavonifractor plautii*	11.61	11.18	19.29	6.31
	*Klebsiella pneumoniae*	6.53	2.02	8.34	0.00

## Discussion

We have demonstrated here that gut microbiome characteristics of trauma survivors were associated with the development of APNS, in this pilot longitudinal study of individuals enrolled in the ED after trauma exposure. Our analysis first suggested that overall inter-individual differences in gut microbiome taxonomy accounts for a substantial fraction (20-48%) of the differences in APNS outcomes. This finding is similar to what is attributable to known APNS outcome-associated clinical and demographic covariates (e.g., sex and race/ethnicity) [45]. We leveraged ML modeling to identify specific bacterial species and microbial encoded metabolic pathways with previously established pro- and anti-inflammatory properties that are predictive of APNS development. Our analysis highlights the most prevalent APNS outcome-associated gut microbiome-encoded pathways are those leading to the biosynthesis of arginine, ornithine, and citrulline. These pathways can possibly affect global arginine levels in the body, a biomarker that has been previously associated with PTSD [46]. To our knowledge, ours is the first study to longitudinally evaluate the link between the gut microbiome and APNS outcomes while providing mechanistic links along the microbiome-gut-brain axis.

Our initial analyses by simple linear mixed-effect models demonstrated the significant contribution of microbiota to APNS outcomes in comparison to clinical covariates and demographic characteristics. The proportion of the gut microbiome accounting for variability in each APNS outcome (26-48%) is of the same order of magnitude as demographic characteristics (10-56%) that are already known to associate with each outcome [45]. However, this analysis required transformation of microbiome abundance variables and the use of a two-tiered approach to select outcome-associated features, which is suboptimal. Although common, this approach neither accounts for the inter-personal variation of the microbiome nor evaluates the effect of microbial species together [47].

While there is evidence for dramatic changes in the gut microbiome after trauma within 72 hours [48], almost no research describing long-term changes in gut microbiota after acute trauma currently exists. One study on PTSD in frontline healthcare workers suggests that long-term gut microbiome dysbiosis induced by stress is sustained for months and predisposes individuals to recurring PTSD [49]. Thus, we built our pipeline assuming the microbiome at our sampling point is representative of the patient’s microbiome at the longitudinal post-trauma outcome measurement points, which we hypothesize associates with target outcomes of developing APNS in prolonged periods after trauma.

ML models such as Mixed-Effect Random Forest (MERF) models are capable of accounting for interpersonal variation, evaluating microbial abundances in the context of the entire microbiome, and, importantly, because no scaling is required, it can be used with different data types (e.g., categorical, numerical, proportion, etc.) [33]Our MERF models identified more species as significant contributors to our outcomes than the linear models and microbes previously associated with neuropsychiatric disorders, specifically *Bifidobacterium*, *Alistipes*, and *Flavonifractor* species. Further, the model identified *F. plautii, E. eligens, E. ramulus, R. homins, C. sybiosum*, and *Blautia* species which are members of the *Clostridium* Cluster IV and XIVa, two clusters of known beneficial bacteria for inflammatory bowel disease and gut health [50–52]. Several of these are short-chain fatty acid producers and have been associated with reduced inflammation in humans [53, 54]. Additionally, they are known to promote several anti-inflammatory immune signatures, such as regulatory T-cell expansion in vivo [55, 56].

Our models based on species abundance identified *Bifidobacterium* species and *Flavonifractor plautii* as the most important factors for predicting all three APNS outcomes. Specifically, we found higher abundances of *B. adolescentis, B. longum*, and *B. bifidum* were associated with higher PTSD and depression scores, which contradicts some literature regarding positive relationships between *Bifidobacterium* spp. and improved neuropsychiatric outcomes [57–59]. Contrary to this, many other microbiome-based studies of neuropsychiatric disorders, including major depressive disorder (MDD), schizophrenia, and anxiety, have failed to find similar associations [60–62]. Indeed, for MDD, various investigators have found both increased and decreased abundances of *Bifidobacterium* spp. to be associated with clinical disease suggesting possibly strain specific effects [50, 51, 62]. Our analysis identifying increased abundances of *Bifidobacterium* spp. as being associated with APNS outcomes adds more contradiction to the literature but highlights the importance of looking beyond species abundances in microbiome studies.

Alone, species-based associative studies can be confounding and limit clearer investigations into biological mechanisms. We have previously shown that also analyzing microbial metabolic pathways can reveal valuable insight into possible biologic impacts of the gut microbiome on clinical outcomes [35, 42]. As with our previous work, our combined analysis approach here, leveraging metagenomic sequencing and ML-based pipelines on both species’ abundance and metabolic pathways, identified a microbially-based signature within the biosynthesis of arginine/citrulline/ornithine as highly informative of the development of all three APNS outcomes. This is the first study to support the existence of microbially produced metabolites acting through the microbiome-gut-brain axis with APNS attributable outcomes.

PTSD patients have been shown to have decreased levels of arginine and increased levels of ornithine and citrulline in peripheral blood [46]. The ratio of arginine to its two main catabolic products, ornithine, and citrulline, is used as a readout of nitric oxide (NO) capacity and is referred to as the global arginine bioavailability ratio (GABR) [52]. Bersani et al. sought to examine NO production in PTSD patients via the GABR as arginine is the sole nitrogen source of NO synthesis. They found that GABR was negatively correlated with PTSD, which they reasoned was indicative of dysfunctional NO synthase. The directionality observed by Bersani and authors mirrors the directionality of arginine-related metabolic pathways in our analysis. Altered arginine metabolism has also been implicated in other neuropsychiatric disorders, such as schizophrenia and MDD [53, 54]. The influence of altered arginine metabolism is likely multidimensional and spans multiple mechanisms of action, including through arginine vasopressin or NO [55, 56]. Identifying microbial sources of these potential mechanisms in specific neuropsychiatric conditions is an important first step towards enabling research on interventions or therapies.

In our species trained MERF pipeline, *Flavonifractor plautii*, a flavonoid-degrading bacterium often found in the human gut microbiome, was the only species among the top 5 contributors to all three APNS outcomes that affect GABR. This genus contributes to the L-arginine biosynthesis pathway via ornithine in those with PTSD and via acetyl in those with PTSD or depression. From our metabolic pathway models, the contribution of *Ruminococcus*, *Alistipes* and *Flavonifractor* genera to L-arginine biosynthesis via ornithine was identified as increased in individuals with PTSD or depression. Although the contribution of these three species were not ranked as highly as *Bifidobacterium* spp. in our species trained MERF model, by analyzing microbial metabolic pathways, we see that their roles in PTSD and depression may be far more significant.

Our findings also provide an added layer of contextual insight into seemingly contradictory findings from prior research on this topic. In addition to being increased in Crohn’s disease, the *Ruminococcus* genera have been identified as decreased in those with MDD in multiple studies [59, 63]. Although we find *R. gnavus* abundance increases with depression score, the contribution of the *Ruminococcus* genera to arginine biosynthesis through the acetyl cycle (ARGSYNBSUB-PWY) and via ornithine (ARGSYN-PWY) was lower in those with depression compared to those without, implying species specific effects may also be at work. Likewise, *Alistipes* have been found to be both increased [63] and decreased in patients with MDD [59, 64] as compared to healthy controls, suggesting genus-based examination of *Alistipes* may not be sufficient. *A. finegoldii* and *A. indistinctus*, were both identified by our microbial model as informative of PTSD score yet with opposite directions. Furthermore, *A. finegoldii* abundance was positively associated with PTSD scores and specifically had a higher contribution to L-citrulline biosynthesis in those with PTSD or depression. We also found that the Alistipes genera contributed more to ornithine-related pathways in those with PTSD and depression. Thus, by combining analyses of species abundance with microbial metabolic pathways, we can better dissect the functional contributions of the microbiota.

The emerging role of polyamines in neuropsychiatric disorders opens a door to a better understanding of the complex pathophysiology of these disorders. The ability of microbiota to produce polyamines and known associations between polyamine-producing microbes and neuropsychiatric disorders highlight the importance of the microbiome-gut-brain axis in human health. Our novel finding of the gut microbiome contributing to alterations in GABR pathways in our studied outcomes is the first direct mechanistic link between the gut microbiome and APNS. This result may provide some indirect evidence of a biological link for APNS along the microbiome-gut-brain axis via microbially generated metabolites.

### Strengths and Limitations

We are the first to delve into the predictiveness of the microbiome to the core components of APNS. Although the sample size of our study was limited, our employment of ML methods and metagenomic profiling enabled us to maximize the utility and richness of data from the samples we had. Furthermore, the AURORA (parent) study ensured a comprehensive, thorough, and standardized system of scoring APNS outcomes longitudinally. Our sub-study involved a single time point collection for every individual and the assumption that the microbiome remains stable over the 2-12 weeks’ time-period post trauma. We acknowledge that this sampling strategy is not ideal and that we would have gained better resolution with having samples at each visit. However, the microbiome tends to stabilize within two weeks event post major perturbations including infection, diet, and antibiotics [65–67]. Therefore we feel that a month after trauma exposure the microbiome have reached a stable yet trauma-shaped state and that the within individual variability that could occur among the different time points would be due to day-to-day fluctuations to be a fair assumption. Additionally, we encountered logistical difficulties with recruitment and sample collection due to COVID-19 restrictions resulting in only 106 of the approach 2,097 participants participating in this sub-study. This challenge necessitated the working assumption that the microbiome is stable following trauma. Further, compared to the parent study, our cohort was generally older, male, and non-Hispanic white (Supplemental figure 1). Despite an established high comorbidity between trauma and substance use [68],we did not find any correlation between our outcomes and substance uses in our cohort, but this should be reevaluated in a larger study as usage may affect the gut microbiome. Future work will aim to expand on our preliminary findings with a larger cohort and more sampling time points, including sampling closer to time of trauma.

Although we approached these APNS diagnoses as discrete outcomes, it is well known that there is much overlap between them. Traditional APNS classification evolved from the realms of specific medical specialties and thus are not indexed to specific biological processes or basis [1]. This biologic overlap may be responsible for the overlap in some features identified by our modeling. A study with a larger sample size may be able to tease apart the overlap of these outcomes. Finally, while our study points to a possible role of the microbiome in mediating APNS outcomes, animal models with selective microbiota modulation will be necessary to determine directionality of this phenotype.

## Conclusions

APNS can have devastating long-term consequences for patients who have already suffered trauma, but APNS may be preventable. Our nested study, using a subset of the AURORA cohort, demonstrated the importance of the microbiome in influencing APNS development. While more work is needed, we are the first to describe a possible biologic link between the gut microbiome and post-trauma outcomes through arginine metabolism and global arginine pathways, which have already been associated with PTSD and other neuropsychiatric disorders. This discovery opens avenues for investigating prevention and treatment strategies through both targeted therapies and microbiome-based interventions. Our findings provide some evidence of a biological link for APNS along the microbiome-gut-brain axis via microbially generated metabolites.

### Supplementary information

Supplemental Table 1

Supplemental Figures legend

Supplemental Figure 1

Supplemental Figure 2

Supplemental Figure 3

Supplemental Figure 4

## Data Availability

Microbiome sequencing data is deposited in the Short Read Archive (SRA) under BioProject accession no. PRJNA940405. All data and scripts produced in the present study are also available upon reasonable request to the authors.
