# Supplementary material for: Association between microbiome and the development of adverse posttraumatic neuropsychiatric sequelae after traumatic stress exposure
Source: Transl Psychiatry. 2023 Nov 18;13:354. doi: 10.1038/s41398-023-02643-8 (PMC10657470; doi:10.1038/s41398-023-02643-8)
Supplement: Supplementary file 1 — Supplemental Table 1 [file 41398_2023_2643_MOESM1_ESM.docx]

­­Supplemental Table 1: Demographics of AURORA Parent Study compared to Microbiome Sub-Study

| **Characteristic** | | AURORA Parent Study, N = 2,392^1^  n (%) | Microbiome Sub-Study,  N = 51^1^  n (%) | **p-value**^2^ | |
| --- | --- | --- | --- | --- | --- |
| **Demographics** | |  |  |  |  |
| Gender | |  |  | 0.2 | |
|  | Female | 1,502 (63%) | 25 (49%) |  | |
|  | Male | 885 (37%) | 26 (51%) |  | |
|  | Non-Binary | 2 (<0.1%) | 0 (0%) |  | |
|  | Transgender | 3 (0.1%) | 0 (0%) |  | |
| Age (at enrollment) | | 33 (25, 45) | 52 (36, 60) | <0.001 | |
| BMI | | 28 (24, 34) | 29 (24, 33) | 0.8 | |
| Hispanic | | 270 (11%) | 7 (14%) | 0.6 | |
| Non-Hispanic Black | | 1,187 (50%) | 14 (27%) | 0.002 | |
| Non-Hispanic Other | | 88 (3.7%) | 1 (2.0%) | >0.9 | |
| Non-Hispanic White | | 847 (35%) | 29 (57%) | 0.002 | |
| **US. Geographic Region** | |  |  | <0.001 | |
|  | Midwest Region | 1,009 (42%) | 10 (20%) |  | |
|  | Northeast Region | 941 (39%) | 38 (75%) |  | |
|  | South Region | 438 (18%) | 3 (5.9%) |  | |
|  | West Region | 4 (0.2%) | 0 (0%) |  | |
| **Trauma Event Type (Broad)** | |  |  |  | |
|  | Animal-related | 57 (2.4%) | 1 (2.0%) |  | |
|  | Burns | 11 (0.5%) | 0 (0%) |  | |
|  | Fall < 10 feet or from unknown height | 126 (5.3%) | 9 (18%) |  | |
|  | Fall >= 10 feet | 40 (1.7%) | 4 (7.8%) |  | |
|  | Incident causing traumatic stress exposure to many people | 10 (0.4%) | 1 (2.0%) |  | |
|  | Motor Vehicle Collision | 1,789 (75%) | 28 (55%) |  | |
|  | Non-motorized Collision | 44 (1.8%) | 3 (5.9%) |  | |
|  | Sexual Assault | 13 (0.5%) | 0 (0%) |  | |
|  | Physical Assault | 217 (9.1%) | 0 (0%) |  | |
|  | Poisoning | 2 (<0.1%) | 0 (0%) |  | |
|  | Other | 83 (3.5%) | 5 (9.8%) |  | |
| Self-Reported Perceived Chance of Dying | | 6.0 (3.0, 9.0) | 6.0 (1.0, 8.5) | 0.2 | |
| ^1^n (%); Median (IQR) | | | | |  |
| ^2^Fisher's exact test; Wilcoxon rank sum test; Pearson's Chi-squared test | | | | |  |
