# Supplementary material for: Association between microbiome and the development of adverse posttraumatic neuropsychiatric sequelae after traumatic stress exposure
Source: Transl Psychiatry. 2023 Nov 18;13:354. doi: 10.1038/s41398-023-02643-8 (PMC10657470; doi:10.1038/s41398-023-02643-8)
Supplement: Supplementary file 2 — Supplemental Figures legend [file 41398_2023_2643_MOESM2_ESM.docx]

**Supplemental Figure Legends**

**Supplemental Figure 1.** Histogram depicting the days since trauma exposure when stool samples were collected from participants. The two-, eight- and twelve-week timepoints for APNS outcome measurements are demarcated in dark purple, light blue and orange respectively.

**Supplemental Figure 2.** Root Mean Square Error (RMSE) and correlation graphs for mixed-effect random forest (MERF) models trained with microbial abundance data. Each of the three timepoints were left out and the microbial and clinical covariates for the remaining timepoints were used to train MERF models to predict (A) PTSD raw score, (B) depression score normalized, and (C) somatic symptoms count (yes/no count). Model RMSE for models tested with the labeled timepoint. Actual versus predicted values for (D) PTSD raw score, (E) depression score normalized, and (F) somatic symptoms yes/no count.

**Supplemental Figure 3.** Relative abundance of MERF identified important microbes across PTSD (A), Depression T-Score (B), and somatic symptoms yes/no count (C). For PTSD and depression, points filled blue indicate no diagnosis (PTSD raw score ≤ 31; Depression T-Score < 60 indicating none to mild depression) red indicates PTSD or depression diagnosis (PTSD RS > 31; Depression T-Score ≥ 60 indicating moderate to severe depression.

**Supplemental Figure 4.** Root Mean Square Error (RMSE) and correlation graphs for mixed-effect random forest (MERF) models trained with microbial metabolic pathway data. Each of the three timepoints were left out and the metabolic pathway and clinical covariates for the remaining timepoints were used to train MERF models to predict (A) PTSD raw score, (B) depression score normalized, and (C) somatic symptoms count (yes/no count). Model RMSE for models tested with the labeled timepoint. Actual versus predicted values for PTSD raw score (D), depression score normalized (E), and somatic symptoms yes/no count (F).
