# Supplementary figures and images for: Association between microbiome and the development of adverse posttraumatic neuropsychiatric sequelae after traumatic stress exposure
Source: Transl Psychiatry. 2023 Nov 18;13:354. doi: 10.1038/s41398-023-02643-8 (PMC10657470; doi:10.1038/s41398-023-02643-8)

# Fecal Sample Collection

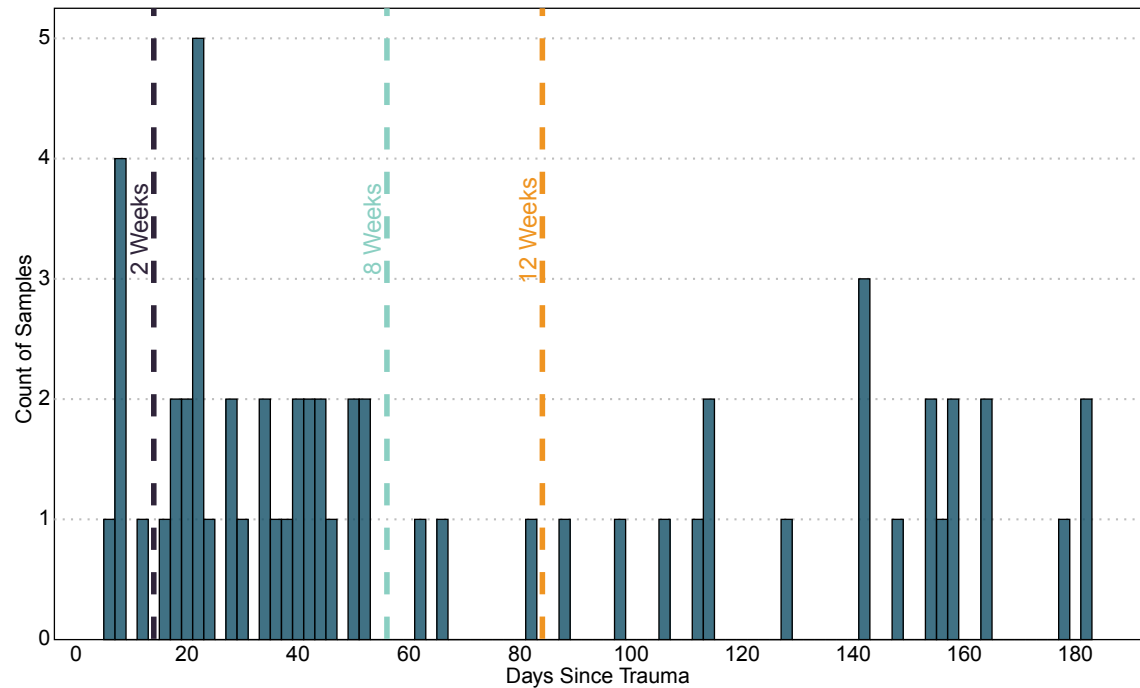

Supplement: Supplementary file 3 — Supplemental Figure 1 [file 41398_2023_2643_MOESM3_ESM.pdf]

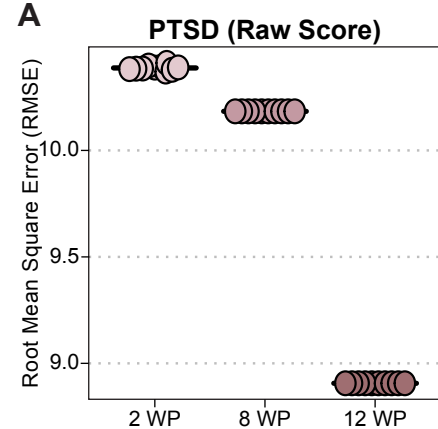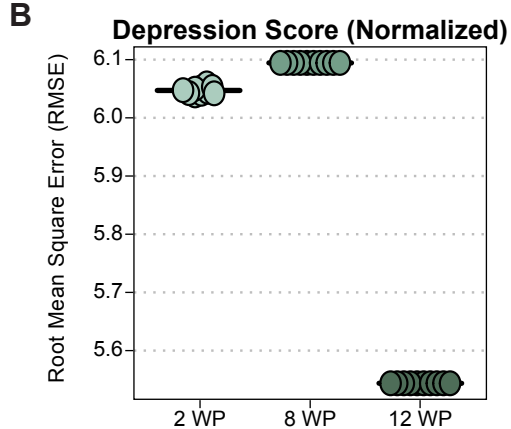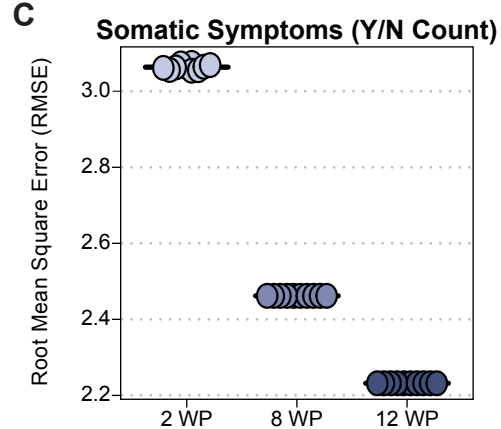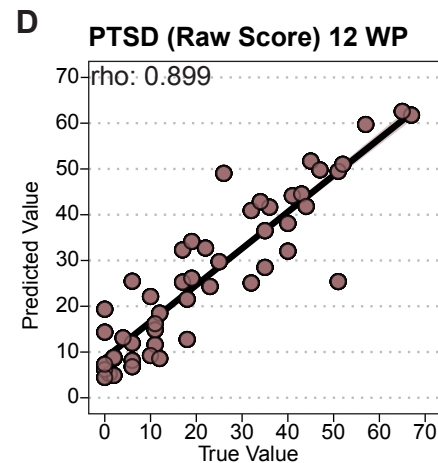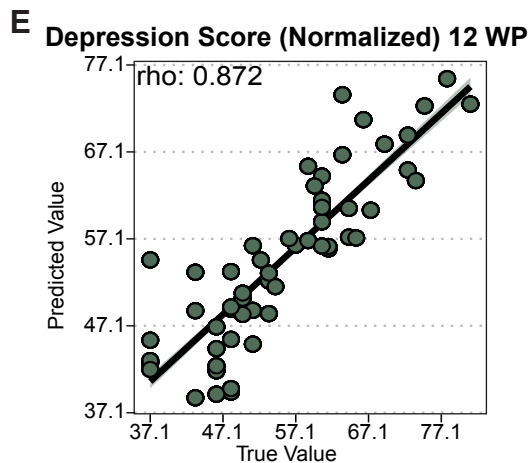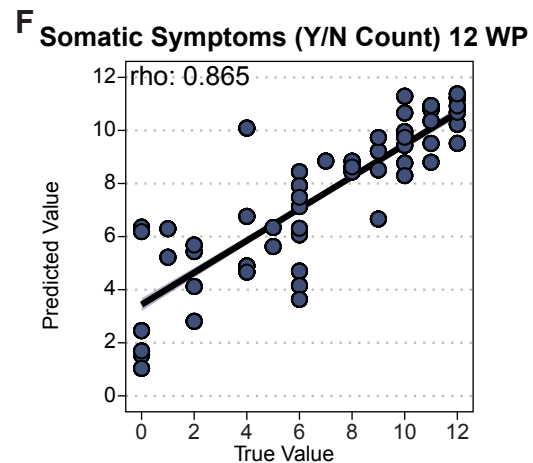

Supplement: Supplementary file 4 — Supplemental Figure 2 [file 41398_2023_2643_MOESM4_ESM.pdf]

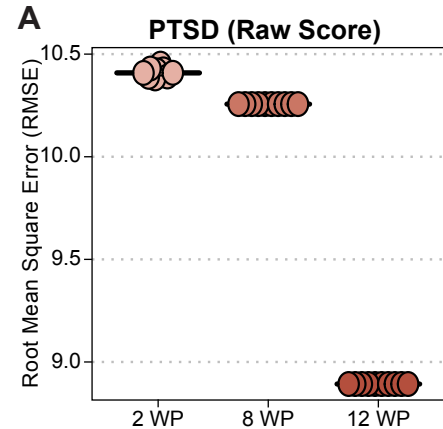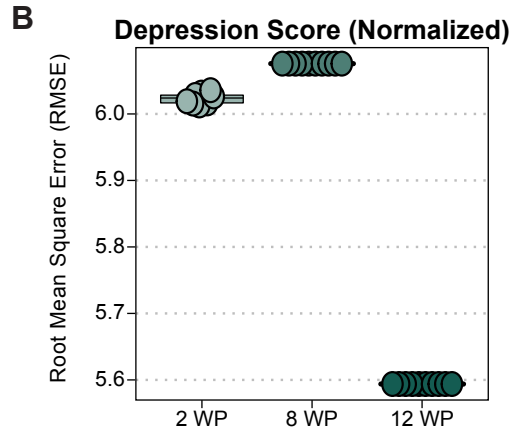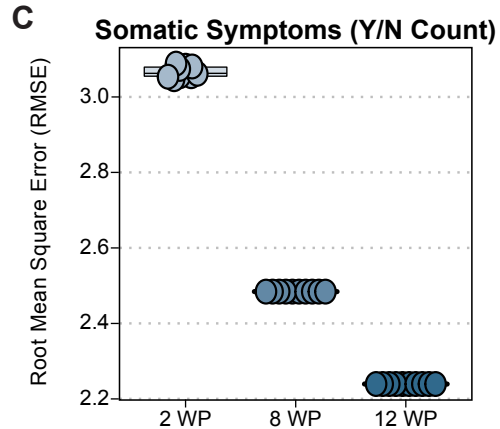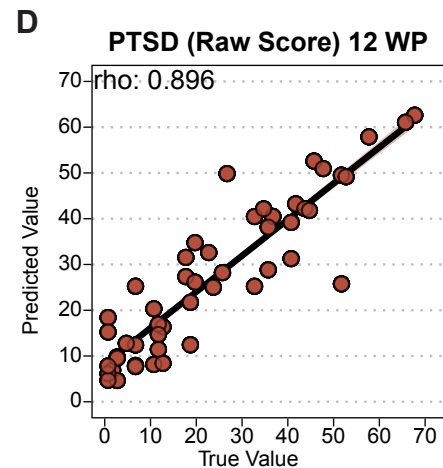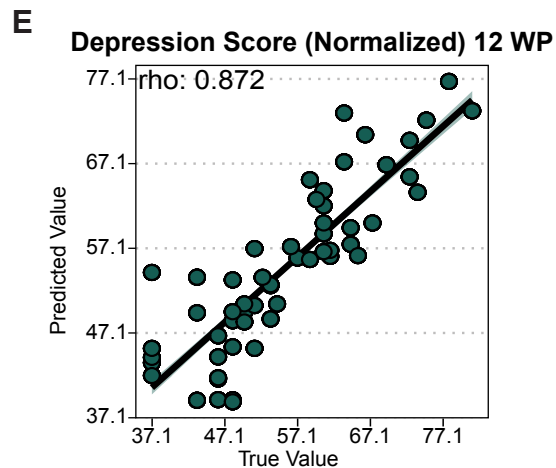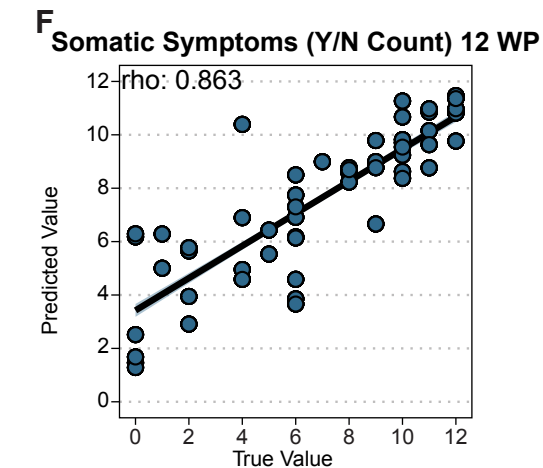

Supplement: Supplementary file 5 — Supplemental Figure 3 [file 41398_2023_2643_MOESM5_ESM.pdf]

**A**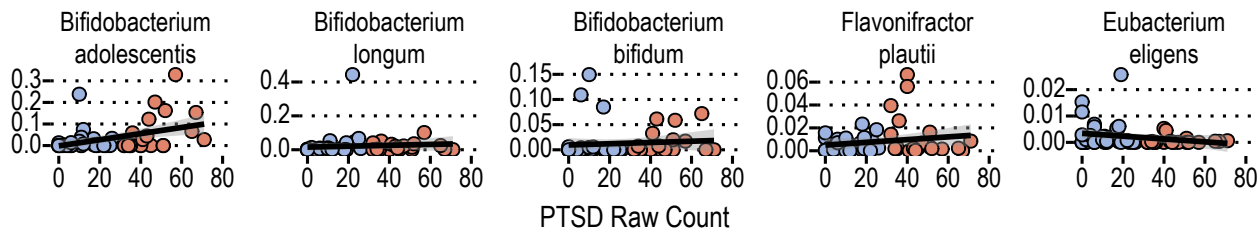**B**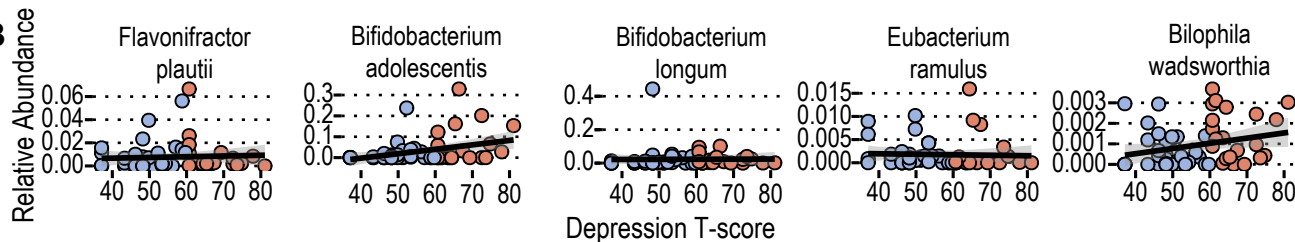**C**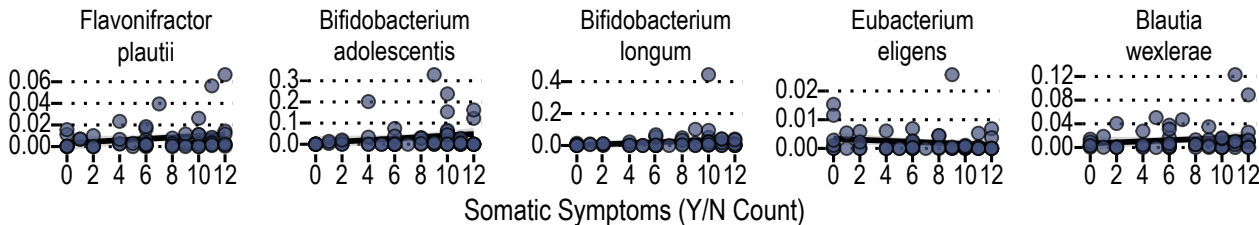

Supplement: Supplementary file 6 — Supplemental Figure 4 [file 41398_2023_2643_MOESM6_ESM.pdf]
